# Smart Hydrogel Swelling State Detection Based on a Power-Transfer Transduction Principle

**DOI:** 10.1021/acsapm.4c00808

**Published:** 2024-04-23

**Authors:** Benozir Ahmed, Christopher F. Reiche, Jules J. Magda, Florian Solzbacher, Julia Körner

**Affiliations:** 1 Department of Electrical & Computer Engineering, University of Utah, Salt Lake City, Utah 84112, United States; 2 Department of Chemical Engineering, University of Utah, Salt Lake City, Utah 84112, United States; 3 Faculty of Electrical Engineering & Computer Science, Leibniz Universität Hannover, 30167 Hannover, Germany

**Keywords:** Stimulus-responsive hydrogel, swelling state transduction, electromagnetic power transfer, inductive coupling, in situ analyte monitoring, microcatheter, flexible sensors

## Abstract

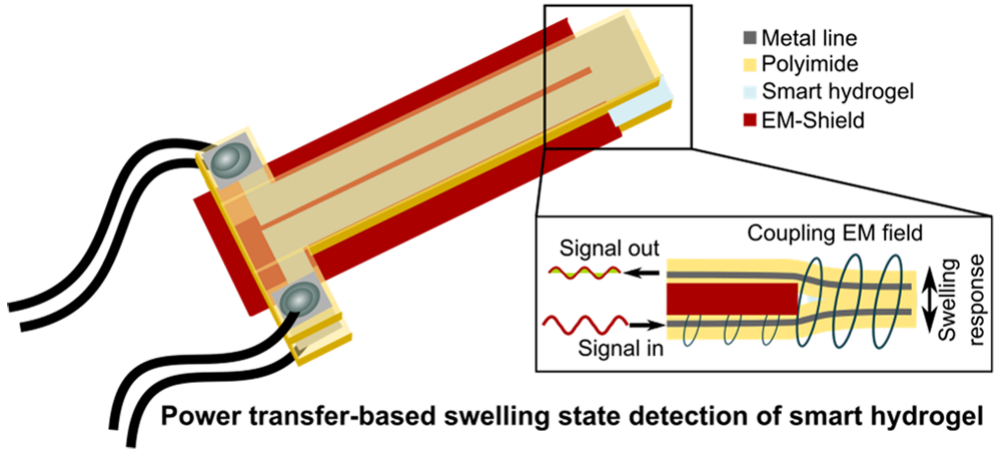

Stimulus-responsive (smart) hydrogels are a promising sensing material for biomedical contexts due to their reversible swelling change in response to target analytes. The design of application-specific sensors that utilize this behavior requires the development of suitable transduction concepts. The presented study investigates a power-transfer-based readout approach that is sensitive to small volumetric changes of the smart hydrogel. The concept employs two thin film polyimide substrates with embedded conductive strip lines, which are shielded from each other except at the tip region, where the smart hydrogel is sandwiched in between. The hydrogel’s volume change in response to a target analyte alters the distance and orientation of the thin films, affecting the amount of transferred power between the two transducer parts and, consequently, the measured sensor output voltage. With proper calibration, the output signal can be used to determine the swelling change of the hydrogel and, consequently, to quantify the stimulus. In proof-of-principle experiments with glucose- and pH-sensitive smart hydrogels, high sensitivity to small analyte concentration changes was found along with very good reproducibility and stability. The concept was tested with two exemplary hydrogels, but the transduction principle in general is independent of the specific hydrogel material, as long as it exhibits a stimulus-dependent volume change. The application vision of the presented research is to integrate in situ blood analyte monitoring capabilities into standard (micro)catheters. The developed sensor is designed to fit into a catheter without obstructing its normal use and, therefore, offers great potential for providing a universally applicable transducer platform for smart catheter-based sensing.

## Introduction

### Smart Catheters

Catheters are widely used in medical procedures and applications such as cardiac surgeries, ultrasound-based tumor treatment, and administration of medication.^1,2^ Equipping these standard medical tools with additional sensing capabilities to enhance and extend their performance is an emerging field of research. Most developments so far focus on improving navigation and handling of catheters inside blood vessels to minimize tissue contact and resulting damage.^3^ This mainly includes different types of force and pressure sensing approaches, such as capacitive and piezoelectric structures on the outside of (balloon) catheters,^4−7^ thin film bending elements protruding from the catheter tip, and integrated optical fibers whose properties are changed upon deformation.^8^ In other cases, temperature sensing and heating capabilities are added to provide not only information about the catheter position but also feedback during ablation procedures in blood vessels,^9^ i.e., enabling multimodal sensing. Furthermore, catheters with integrated optoelectronic capabilities have been reported for in situ tracking of cardiac blood oxygen levels.^10^ Another development are actively controlled elements, which are integrated into the catheter to enable magnetic-based movements such as bending or even microgripper functionality and actuation.^11^

However, to date, there are only very few approaches targeting the creation of a smart catheter for analyte that enables real-time monitoring of fentanyl and propofol levels during surgeries based on an electrochemical principle (voltammetry). Thereby, two different modified carbon paste-based electrodes as well as two Ag/AgCl reference electrodes are incorporated into a microcatheter with 1 mm diameter.^12^

Catheters provide an ideal pathway for biomedical sensor development with the target of (almost) real-time analyte monitoring during and to some extent after medical procedures. However, despite a clearly expressed need for such technologies, current implementations remain sparse and novel approaches need to be developed.^13^ Main challenges in that regard include the sensing principle itself, i.e., the development of suitable target-analyte specific sensing materials and corresponding transduction methods. Within the biomedical context, this is specifically challenging due to the required biocompatibility of all employed materials and components, even if the sensor may only be used during surgical procedures and not implanted for prolonged amounts of time.

Ultimately, the aim of our research is the design of a smart catheter, where this standard medical tool is equipped with sensing capabilities for biomedically relevant analytes. The first step toward this goal, which we report here, is the development of a robust, reliable, and miniaturizable sensor concept, comprising the sensing material and a transduction concept that has a suitable form factor for catheter deployment and consists of potentially biocompatible materials.

### Stimulus-Responsive Hydrogels as Sensing Elements

For this purpose, stimulus-responsive (smart) hydrogels have been chosen as the sensing material, since this specific type of hydrogel is capable of exhibiting a reversible and reproducible volume-phase transition in response to a wide variety of physical and chemical stimuli such as temperature, pH, light intensity, or specific analytes.^14−16^ Similar to their nonresponsive counterparts, smart hydrogels can be tailored to feature mechanical properties akin to biological tissue and can be made from many different base materials, including biocompatible ones. An adjusted stimulus-specific responsiveness can be achieved by employing selective functional groups, aptamers, or molecular imprinting methods,^17−19^ which opens many new application possibilities where the swelling changes in dependence on a specific analyte concentration or stimulus strength can be harnessed.^15,20−26^

However, while hydrogels in general are frequently used in biomedical contexts due to their aforementioned favorable properties, e.g., as scaffolds in tissue engineering, location-specific drug delivery, coating material for implants or wound patches,^27−29^ their smartness potential for sensing applications is scarcely explored to date.

The main reason for this is the lack of suitable transduction techniques that enable a robust and reliable transformation of the hydrogel’s volume change into an electric signal. A corresponding sensor (smart hydrogel sensing element plus transducer) needs to ensure a stable and reproducible detection of the hydrogel’s swelling state even for small changes and in the presence of noise and cross-sensitivities, especially common in biomedical settings. Furthermore, for such use cases, the transducer and complete sensor need to be biocompatible as well.

### Transduction Concepts for Smart Hydrogel Swelling States

These challenging requirements have been addressed by a variety of transduction principles which include optical, mechanical, and electrical approaches as well as combinations thereof.^22,30,31^

In optical methods, the hydrogel serves as an optical element whose properties are altered by swelling or shrinking. Detection concepts include fluorescence, photonic crystal structures, and periodic optical gratings.^32−41^ Challenges in biomedical contexts include complex transducer design, algorithm development for stable signal extraction with high signal-to-noise ratio (SNR), and micro/nanopatterning of hydrogel sensing elements.

Purely mechanical concepts are mainly based on cantilever sensors whose static bending or dynamic oscillation properties are altered when the attached hydrogel swells or shrinks (increase or reduction of mass). Most applications rely on the static bending change, also termed as “surface stress-based sensors”, and an optical readout thereof.^42−45^ The latter requires a rather bulky readout that is difficult to miniaturize and, furthermore, a clear optical pathway.

Another recently developed mechanical concept is harnessing ultrasound as a readout for a smart hydrogel membrane forming an acoustic resonator sheet. The transduction relies on the swelling state dependence of the sheet’s resonance frequency where a reduction of the observed reflected wave intensity occurs. This concept has already been demonstrated in *in vivo* test cases for glucose sensing^46,47^ and furthermore been suggested for integration into a catheter.^48^

Transduction concepts that only use the electrical properties of the hydrogel are rare and mainly rely on conductometric measurements based on interdigitated electrodes (IDEs). Thereby, the hydrogel is placed on top of the IDE and its swelling/shrinking changes the conductivity within the IDE, resulting in a change of impedance or output voltage.^49^

Much more common are approaches based on the combination of mechanical and electrical effects that can be categorized into capacitive, inductive, and piezoresistive. In all cases, the hydrogel’s volume change induces a mechanical deformation on an electrical element, leading to an electric output signal. For capacitance-based concepts, mainly two configurations are reported: the hydrogel is either sandwiched between two capacitor plates^50,51^ or placed on top of the complete microcapacitor.^52,53^ In the former case, binding of the target analyte alters the electric permittivity of the hydrogel and/or the spacing between the electrodes, resulting in a change of the capacitance. This has for example been demonstrated for a glucose-sensitive hydrogel relying on the change of internal charge distributions when glucose molecules bind with boronic acid integrated into the polymer structure.^50,51^ In the second capacitor setting, the hydrogel is attached to a flexible capacitor plate that is placed above a rigid second plate at a defined initial spacing. The volume change affects the distance between the two electrodes (increased deformation of flexible plate).^52,53^ Regardless of the specific transducer geometry, the resulting change of capacitance is usually detected by a frequency shift of an integrated resonance circuit, spanning frequency ranges from a few tens of kHz up to GHz.

As an alternative to capacitive effects, inductive coupling is used as well. Thereby, the transducer configuration is very similar to the hydrogel being placed between two plates. In contrast, these contain two-dimensional inductor structures and the transduction of the hydrogel’s swelling state is based on the change of mutual inductance (and partly also capacitance as these are linked), also read out by resonance circuits.^54^

Furthermore, a bending sensor concept that is harnessing the general electric impedance change of a deformed thin film sensor with an embedded metal layer has been reported.^55^ The design of this transducer structure served as a starting point for the work reported here.

Another very common transduction method is piezoresistive pressure sensing. Thereby, the hydrogel is placed in a cavity closed with a thin silicon membrane with integrated piezoresistive elements. Contact between solution and hydrogel is achieved with a solution inlet/outlet at the bottom of the cavity. The hydrogel swelling exerts a force on the membrane, and the deformation is transformed into an output voltage via the piezoresistors. This approach has frequently been used in the characterization of hydrogel materials^56−58^ and been modified, e.g., for active control settings with bisensitive hydrogels,^59^ or by placing the hydrogel on top of the bending membrane instead of into the cavity to increase contact with the target analyte when applied for gas sensing.^60^

All of these mechanoelectrical concepts offer the potential for miniaturization and sensitive detection of the hydrogel’s swelling state. Challenges include the aspects of biocompatibility (e.g., in case of piezoresistive pressure sensing), contact between hydrogel and target analyte which has a significant influence on response time and therefore real-time capabilities, and proneness to noise and interferences, specifically for the high-frequency readout schemes.

This overview highlights the main developments in transduction concepts for smart hydrogels. Different physical principles, all with their advantages and limitations, have been explored, and so far, only very few of them are suitable for integration into a smart catheter for analyte monitoring in blood. The main persisting challenges are (i) the form factor and limited space within a catheter, especially if it is still to be used for drug delivery in parallel to the sensing capabilities, (ii) robustness and stability of the sensor output signal in the bioenvironment that can induce significant noise and disturbances, and (iii) biocompatibility of the sensing material and transducer.

The presented research focuses on addressing these considerations and requirements through a novel thin-film power-transfer-based transduction principle, which belongs into the above-described group of mechanoelectrical concepts. The key features of our approach are the versatility of sensor geometry and design, its minimal dimensions which only fill less than 10% of the total volume of a 1.5 mm diameter medical microcatheter, the stability and reproducibility of its sensing performance, and the potential platform capability.

In the following, we present the proof-of-concept studies conducted for two different types of hydrogel and analyte, namely, glucose and pH, and discuss relevant sensor properties. The aim is to demonstrate the feasibility of the transduction principle as a first step toward a future smart catheter for in situ and real-time blood analyte monitoring.

## Materials and Methods

### Sensor Concept

#### Transduction Principle

**Figure 1:**
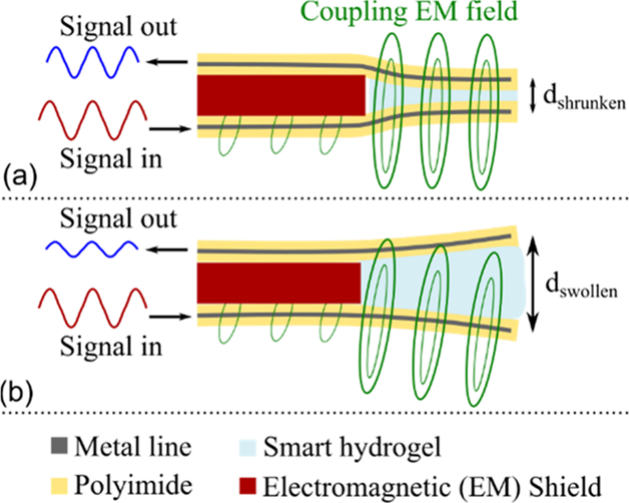
Illustration of the concept for deformation-modulated power transfer for transduction of a smart hydrogel’s swelling state with (a) shrunken and (b) swollen hydrogel. The volume change alters the amount of electromagnetic field energy that is transferred from the sender (lower transducer part in both subfigures) to the receiver (upper part), resulting in a change in magnitude and phase of the measured output signal.

The presented transduction concept for the swelling state of a smart hydrogel sensing element is based on a deformation-modulated change of the transferred power between two conductive structures. Figure 1 depicts the basic principle: the smart hydrogel is sandwiched between two metal strip lines embedded in a polymer thin film. One transducer part (the lower one in Figure 1a, denoted *sender* in the following) is driven with an alternating current signal that generates an electromagnetic (EM) field around it. The power flow (intensity and direction) of this field is described by the Poynting vector and in free space it is rotational symmetric around the field generating element with deceasing magnitude for increasing distance from the sender.

The second transducer part (upper sheet in Figure 1a, denoted as *receiver* in the following) is penetrated by the EM field from the sender, and the transferred power induces a voltage in the receiver that can be measured as an output signal.

The smart hydrogel modulates the distance between sender and receiver through its volume change, thereby affecting the amount of transferred power and hence altering the magnitude and phase of the receiver output signal. This is illustrated for two different hydrogel swelling states in Figure 1a,b.

Note that the hydrogel in general will change not only the distance between sender and receiver but also the orientation of the sheets (angle), depending on the stiffness of the films in relation to the hydrogel thickness. However, for the basic principle the specific cause (spacing and/or angle) for a position change of the two transducer parts is irrelevant, as long as it is reproducibly induced by the hydrogel’s response to the stimulus/analyte. Furthermore, it is a prerequisite for the power transfer functional principle that the hydrogel thickness and swelling change be designed to ensure that the receiver is within the region of the electromagnetic field generated by the sender.

If properly calibrated, the obtained receiver output voltage is then related to the stimulus concentration or strength, causing the volume change of the hydrogel.

#### Sensor Design and Fabrication for Proof-of-Concept Investigation

##### Design Rationale

**Figure 2:**
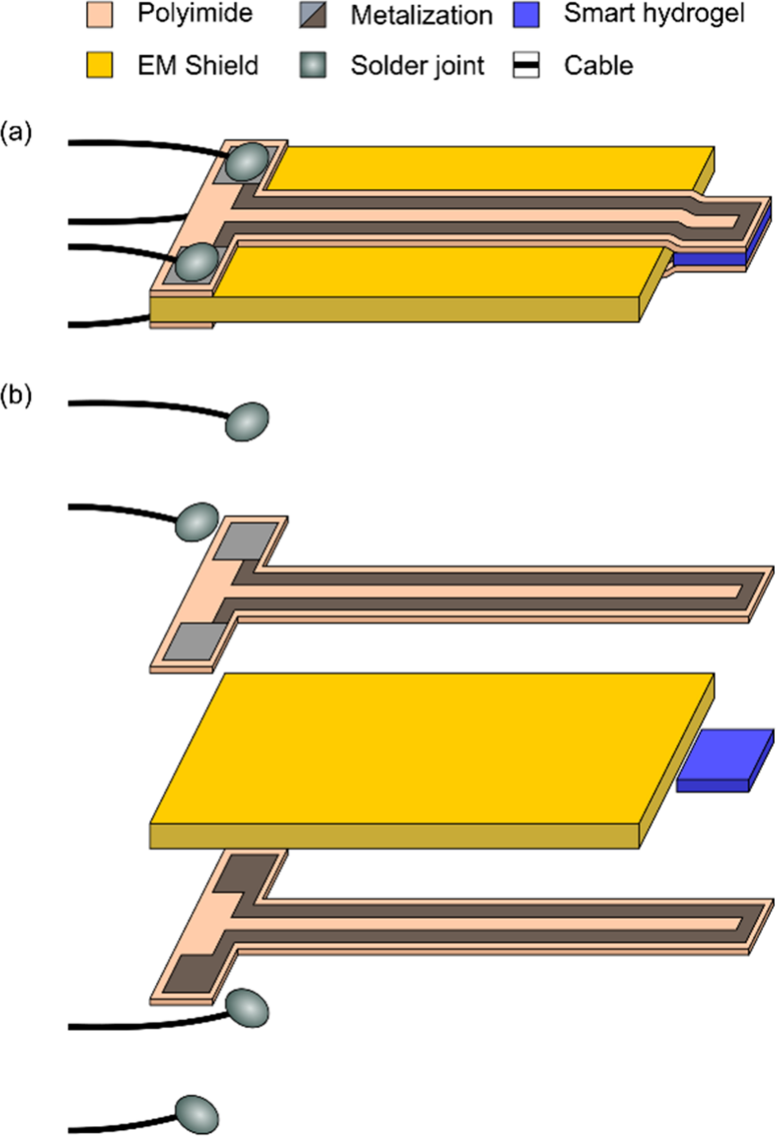
Principle sketch of the power transfer-based sensor platform (not to scale): (a) assembled sensor structure; (b) explosion drawing of sensors parts comprising polyimide encapsulated metal transducer structures, smart hydrogel, electromagnetic (EM) shield, and connective cables with solder joints. Note that in both cases, the medical grade epoxy encapsulating the solder joint and bond pads as well as the outer foil shielding protecting the sensor from external electromagnetic interferences are omitted for clarity. An image of an actual microfabricated transducer structure can be found in the Supporting Information (Figure S2).

For the proof-of-concept study, the transducer design schematically depicted in Figure 2 is chosen. It features two sheets that each consist of a single-turn (U-shaped) platinum strip line of 800 nm thickness embedded in the middle of a 6.3 μm thick polyimide (PI) film. The overall dimensions of the PI encapsulation are 15 mm × 480 μm (length, width), and the platinum lines have a line width and interline-spacing of 230 and 10 μm, respectively. The metal loops end in bond pads where the PI encapsulation is removed to enable soldering of connection cables.

The described geometry has been chosen based on previous investigations of mechanical stability and the relation between transducer stiffness and hydrogel thickness,^61^ and with respect to our target application, the integration into a catheter (leading to the elongated shape). Furthermore, the aim of the presented investigation is the principle evaluation of the feasibility of the power transfer concept for smart hydrogel swelling state detection. Hence, the metal strip line is designed as a simple U-shape to minimize the current pathway and resistance, thereby maximizing the current flow, which is necessary for efficient power transfer. Furthermore, this design reduces secondary and parasitic electric effects that would likely occur in more complex geometries.

However, increasing the number of turns (e.g., in a meander) could potentially increase the power transfer, but this would also increase the current path and therefore the thin-film resistance, as well as proneness for interferences and noise. Depositing a thicker layer of platinum could reduce the resistance but could increase the stiffness and consequently the transducer’s ability to deform in response to changes in hydrogel volume. Therefore, one needs to carefully consider and balance all aspects for adapting the concept for specific future applications.

##### Transducer Fabrication

The individual transducer sheets are fabricated by standard microfabrication techniques (the details are outlined in the Supporting Information). After fabrication, they are assembled into the complete transducer structure depicted in Figure 2. Thereby, the PI sheets are connected back-to-back with an electromagnetic shield between them except for the tip region where the smart hydrogel is located. Coaxial cables (RG174) are soldered to the bond pads, and the connection points are stabilized and insulated by an additional layer of medical grade epoxy (Hysol M-21HP, Loctite).

To minimize crosstalk along the length of the transducer parts and restrict the electromagnetic interaction to the tip region, an electromagnetic shield is placed along two-thirds of the total length, starting from the bond pads. This is achieved by attaching the two transducer parts to a gold sputtered glass slide (1 mm glass with 200 nm gold) that acts as a stabilizing substrate during the experiment and provides the electromagnetic shielding between the parts due to a gold layer. To protect the sensor from external electromagnetic interference, the respective other side of each transducer part is covered with a 150 μm thick aluminum foil sputter coated with gold.

##### Smart Hydrogel Fabrication

The smart hydrogel sensing element of 400 μm thickness and 480 μm width is polymerized between the PI sheets on the remaining 5 mm unshielded tip region. The details of hydrogel fabrication and secure attachment to the polyimide are described in the Supporting Information. For the presented studies, synthetic acrylamide-based glucose- and pH-sensitive smart hydrogels are employed. Please note that due to the different thicknesses of the hydrogel and the glass substrate, the transducer sheets are slightly bent at the tip region where the hydrogel is placed as indicated in the sketch in Figure 2.

The fully assembled sensor consists of two PI sheet transducers (sender/receiver), the glass-based shielding, and smart hydrogel sensing element as well as cable connections to the measurement equipment (see Figure 2).

### Experiment Design

To investigate the potential of the power transfer concept for smart hydrogel swelling state detection and analyze respective sensor properties, two different exemplary hydrogels with glucose and pH sensitivity, respectively, are used as sensing elements. The corresponding details of the measurement setup and test protocols are outlined in the following.

#### Measurement Setup

**Figure 3:**
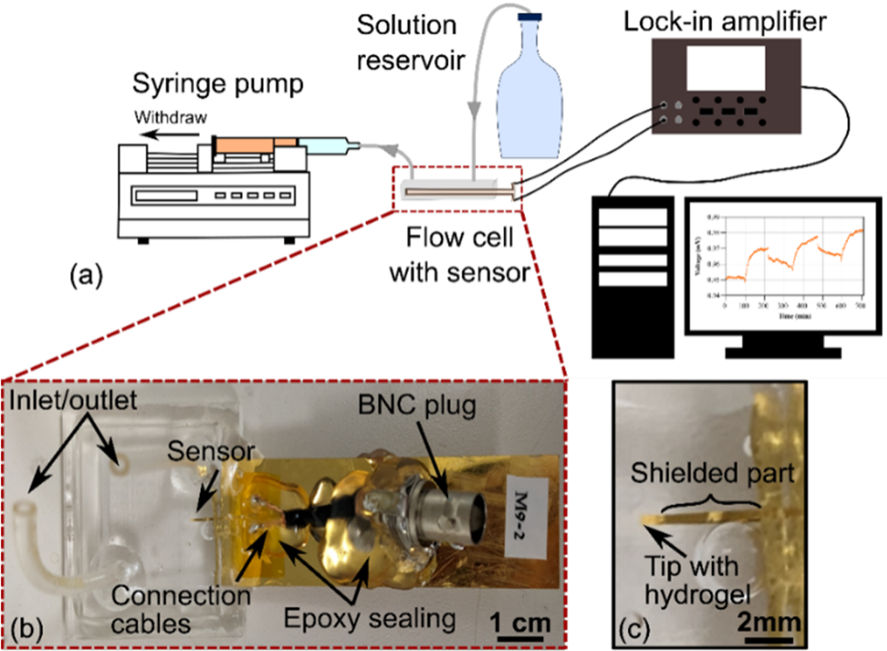
Experimental setup for characterization of the power transfer-based sensor: (a) overview, (b) flow cell with sensor and cable connection to BNC plugs, and (c) magnified image of the sensor. Note that in (b) only one BNC connector is visible as the other is underneath the golden-colored shield.

The measurement setup comprises a flow cell for immersing the sensor in the desired liquid-based stimulus, electrical readout equipment, and a syringe-pump system for liquid exchange (Figure 3).

The flow cell is made from two 1/4 in. thick acrylic plexiglass sheets, where the bottom one contains a laser-cut well to hold the sensor and solution (capacity of 0.39 mL). The top one is equipped with an inlet and outlet where silicone tubes (1/8 in. (3 mm) × 3/16 in. (5 mm), inner and outer diameters, respectively) are attached.

During assembly, the sensor is placed in the well in such a way that only 10 mm of the total 15 mm sensor length is ultimately enclosed in the flow cell. The remaining 5 mm containing the bond pads and cables was kept outside the cell to prevent any contact with fluids.

The plexiglass parts along with inlet and outlet tubes are assembled and glued together with medical grade adhesive (Loctite AA 3321, Henkel) to create the flow cell prior to sensor placement. Then the sensor is positioned inside the cell and glued in place with the same medical grade adhesive. Note that the sensor is permanently fixed in the flow cell and cannot be removed after measurement. Hence, an individual cell must be used for each studied sensor.

Inlet and outlet are connected to a solution container and a programmable syringe pump (model 780212, KD Scientific Inc.) for automated flow control and solution exchange. The two cables of each transducer part are soldered to 50 Ω BNC female plugs that are connected to a lock-in amplifier (UHFLI, Zurich Instruments) by coaxial cables with 50 Ω characteristic impedance and BNC-male ends. The lock-in amplifier is connected to a computer for data recording.

To ensure stable and reproducible measurement conditions for each experiment and for prolonged periods of time, the following measures are taken:

- Only two-thirds of the sensor length are inserted in the cell while the rest remains outside as described above, to avoid contact between the analyte solution and the epoxy sealing of the cable connections. Ions in the solution can degrade the epoxy and therefore alter the electrical properties of the solder joint over time and cause a drift of the output signal.
- Thorough inspection of the flow cell with an optical microscope was done prior to starting an experiment to check for any damage in the sealing and the presence of air bubbles inside the cell. If bubbles are observed, they are removed by flushing the cell with saline solution at high flow rates.
- Right before starting the measurement, the complete flow cell is covered with aluminum foil acting as an electromagnetic shield. This is connected to the ground shield of the BNC plug of the coaxial cable with a Kapton tape which in turn is connected to the ground of the measuring instrument (lock-in amplifier) to reduce any external interference. Additionally, the connection cables are secured with tape to prevent any sudden changes in the signal due to mechanical vibrations.

#### Study Solutions

The necessary solutions are prepared in individual bottles with a base of 1× phosphate buffered saline (PBS) solution (6506-1L, OmniPur). For glucose studies, glucose powder (dextrose, Sigma-Aldrich) is added to 1× PBS in varying amounts (0.90 g, 0.54 g; 1.08 g; 1.62 g; 2.16 g) to reach concentrations of 5 mM (for functionality test) and 3 mM, 6 mM, 9 mM, and 12 mM (for step and reset test), respectively. Note that “0 mM” represents pure 1× PBS in the following.

The pH solutions are prepared by adding the required amount of 0.1 M NaOH to the 1× PBS base to achieve the desired pH values between 7.4 and 8.2. pH values of the solutions are verified with a benchtop pH meter (PC700, Oakton).

During the experiments, the sensor is subjected to a continuous stable solution flow of 0.1 mL/min controlled by the syringe pump, and solutions are changed by simply switching bottles. To prevent the introduction of air bubbles during solution exchange, the pump is turned off for 30 s when exchanging the container.

#### Sensor Test Protocols

Three different tests featuring varying solution exchange protocols are conducted to characterize sensor performance and determine the relevant parameters. For all three experiments, the solution exchange interval between each step is set to 6 h. To ensure identical and stable initial measurement conditions, the sensor is always immersed in 1× PBS for 24 h prior to an experiment. Then the cycling for the respective test (described in the Results and Discussion section) is commenced.

##### Functionality Test

The aim of the functionality test is to evaluate the basic feasibility of the concept and determine sensor baseline, drift, reversibility, and reproducibility. Therefore, the sensor is subject to 4 cycles of alternating stimuli concentrations.

##### Step Test

In the step test, the sensor is subjected to stepwise increasing and decreasing stimuli concentrations to determine sensitivity, reproducibility, and detection limits. The respective stimuli concentrations are chosen to reflect the full responding range of the tested hydrogel. One measurement cycle consists of four steps of increasing concentration, followed by the same four steps in decreasing order, and the experiment is conducted for two complete cycles.

##### Reset Test

The reset test is similar to the step test, but the sensor is put back into 1× PBS after each stimulus concentration step. This allows us to evaluate reversibility and hysteresis, i.e., studying if the stimulus concentration influences the sensor’s ability to go back to its initial state. In this test, one measurement cycle comprises three stimulus concentrations in increasing order with a 1× PBS step between each one. The complete experiment is conducted for two cycles.

#### Data Recording

During the experiments, the change of the output voltage resulting from hydrogel swelling and corresponding effect on the transferred power is recorded with the lock-in amplifier software (LabOne, version 20.02, Zurich Instruments) at a sampling rate of 1.67 samples/s at 13 MHz operating frequency and stored as.csv files. The operating frequency is chosen based on frequency spectra of the receiver for sensor immersion in 1× PBS and 5 mM glucose solution. At 13 MHz, a maximum change in voltage occurs (Figure S4 in the Supporting Information) and therefore this frequency is chosen for all measurements

The amplitude of the input sinusoidal signal is set to 10 mV. The input and output impedance of the lock-in amplifier were set to 50 Ω to match the impedance of the coaxial cables.

## Results and Discussion

To evaluate the feasibility of the power transfer concept for the transduction of a smart hydrogel’s swelling state, a glucose-sensitive hydrogel and a pH-sensitive hydrogel (denoted as GSH and PSH, respectively) are studied. They constitute two different stimuli and corresponding volume change strengths. The hydrogels are fabricated on the transducer tip, and the above-described test protocols are carried out for each sensor.

In view of the target application of analyte monitoring in blood, the respective glucose and pH test values are chosen based on conditions in human blood and, for glucose, on previously reported comprehensive analysis of the used GSH.^56,57,62^ Note that the aim of these proof-of-concept experiments is the demonstration of the transduction principle and not the characterization of the hydrogel. The latter serves simply as an exemplary and well-characterized sensing material for test purposes. Hence, the test ranges are set based on the known and precharacterized values for the materials.

### Glucose-Sensitive Hydrogel (GSH)

The physiological range of interest for glucose sensing is 0–20 mM.^63^ For our investigation, glucose concentrations from the midrange are chosen for evaluation of the transduction detection concept. Concentrations are alternated between 0 mM (1× PBS only) and 5 mM glucose in the functionality test. In step and reset tests, concentrations of (0; 3; 6; 9) mM are used with the additional 12 mM for the step test.

**Figure 4:**
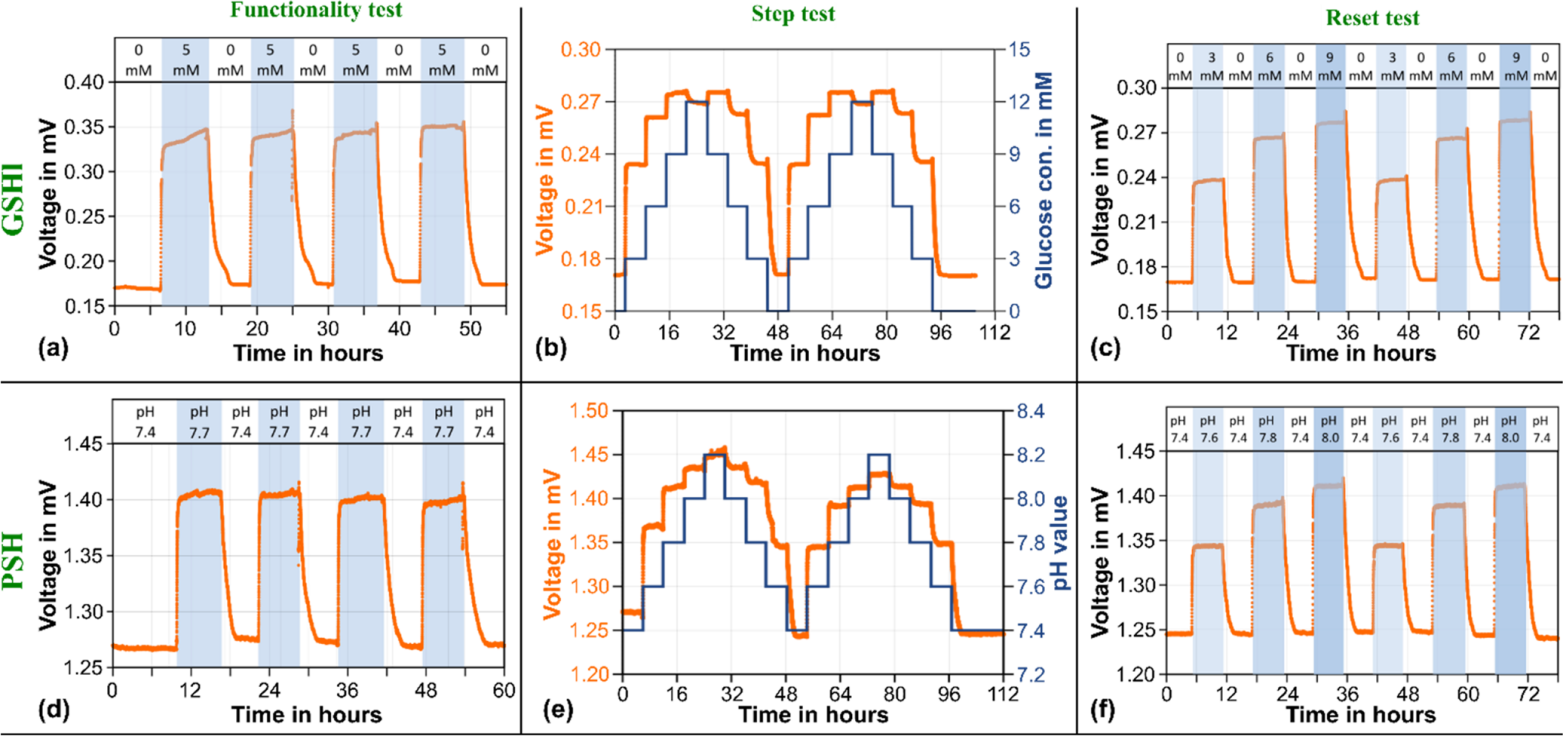
Sensor output voltage for a glucose- and a pH-sensitive hydrogel (top row GSH, bottom row PSH) in (a, d) functionality, (b, e) step, and (c, f) reset test.

The results are depicted in Figure 4. For all three test scenarios, the sensor output voltage shows a stable and reproducible response and no baseline drift. For step and reset tests (Figure 4b,c) the dependence of the output voltage magnitude on the glucose concentration is clearly visible.

The overall sensor output voltage is in accordance with the expected behavior of the used hydrogel: The GSH is shrinking with increased glucose concentrations as charges of the polymer backbone are altered due to the binding of glucose molecules to boronic acid. Shrinkage of the hydrogel reduces the separation between the transducer part, which increases the amount of power transferred from the sender to the receiver and consequently results in an increased output voltage. Vice versa, decreasing glucose concentrations lead to an increase of charge-induced repulsive forces within the polymer structure, cause the hydrogel to swell, and lead to a decrease of transferred power and a reduced receiver output voltage as the transducer parts are moved farther apart. This behavior is consistently observed in all experiments.

In general, the sensor equipped with the GSH provides a consistent and reproducible output signal for the various test cases: repeatable alternation between stable voltage values for the functionality test and decreasing delta voltage with increasing glucose concentration in the step and reset test. However, for the highest concentration of 12 mM in the step test, a reproducible reversed response is observed (a decrease in voltage instead of increase). This can be attributed to the hydrogel itself: the used GSH is designed to respond to glucose by the integration of phenyboronic acid, which reversibly forms complexes with diols from the glucose. The phenylboronic acid itself provides negative charges in the polymer backbone. Despite the tertiary amine causing additional positive charges, the net charge of the polymer is negative. Upon binding of glucose, the formed complexes create positive charges, which reduce the electrostatic repulsion forces within the polymer network and cause the hydrogel to shrink. However, when the glucose concentration surpasses 9 mM, the excess amount of available glucose leads to an increased competition for binding sites on the phenylboronic acid. This in turn increases the overall negative charge and causes an increased electrostatic repulsion force, resulting in the reversal of the response and therefore swelling.^46,47,62^

Note that the smart hydrogel composition would have to be tailored if a sensor for a larger glucose concentration range is to be designed. However, this is beyond the scope of the presented research, as the subject of this study is to verify the transduction principle itself, independent of the hydrogel and target application.

### pH-Sensitive Hydrogel (PSH)

For measurements of the PSH, 1× PBS with an initial pH value of 7.4 is chosen to reflect the normal pH of blood. In the functionality test, the pH of this base is alternated between 7.4 and 7.7, and in step and reset test, the value is varied in 0.2 increments up to 8.2 and 8.0, respectively. The used PSH shrinks in response to an increase in pH. The results depicted in Figure 4d–f show an equally stable and reproducible sensor response like the GSH.

Only the first cycle of the step test appears to be a bit noisier, and furthermore, a drop in the baseline after the first cycle compared to the initial condition is visible. This can most likely be attributed to the influence of an undetected bubble in the test chamber. Such results were frequently observed in previous experiments when bubbles appeared in the environment of the hydrogel or transducer. To reduce the chance of this happening, the flow cell design and experimental protocol was optimized to what is presented above. However, even with these optimizations, bubble formation cannot be completely prevented.

For both types of hydrogels and stimuli, all measurements indicate an asymmetry in the response time, i.e., a faster shrinking than swelling and corresponding change of the output voltage signal. This is the expected behavior for the smart polymers used,^46,62,64^ and the results demonstrate that the transduction concept is sensitive and fast enough to capture these effects.

It should be noted that due to the long holding times of 6 h per concentration/condition, the number of total test cycles has been limited to what is shown in Figure 4. These tests already take several days with a maximum of 110 h (corresponding to more than 4 days) for the step test. This is not a long-term study but it indicates that the transducer works stably and reproducibly for prolonged amounts of time without failure. Furthermore, several sensors have been fabricated and characterized with the same protocols and all exhibited a similar stable performance. This is a clear hint for the robustness of the concept.

To enable real-time analyte monitoring in a potential catheter application, it is imperative to optimize the hydrogel sensing element for a faster response. However, this is again a question of material optimization and adjustment. The presented transduction principle itself is clearly suitable for capturing rapid swelling changes. The comparatively longholding time was only selected to achieve an equilibrium state of the hydrogel after each step, avoiding additional effects and ensuring a clear focus on evaluating the features of the transduction principle.

### Control Sensor

To validate that the measured output voltage signal is not caused by the transducer structure itself responding to changes of the stimulus concentration, a nonresponsive control sensor was fabricated and tested with the same glucose concentrations as described in the above test protocols. Thereby, the smart hydrogel was replaced by medical grade epoxy (Hysol M-21HP, Loctite) with the same dimensions as the hydrogel (thickness, 400 μm; width, 480 μm; length, 5 mm). No dependence on the glucose concentration was found in either test, and the output signal remained stable except for random voltage fluctuations. Furthermore, the absolute output voltage is (0.27–0.28) mV, which is very similar to the baseline voltage of the other studied sensors. However, fluctuations of the signal are less than 0.01 mV and do not follow the stimulus change, while the change of output voltage for the active sensors, that is with a smart hydrogel, is in the order of (0.1–0.2) mV (see Figure 4). The data for the control sensor are summarized in the Supporting Information (Figure S5).

From the conducted experiments, it can be concluded that the power transfer-based concept is capable of reliable detection of a smart hydrogel’s swelling state. Based on the measured data, the sensitivity and limit of the detection of the concept can be evaluated as discussed in the following. Note that these considerations encompass the complete sensor, i.e., the combination of measurement equipment, transducer part, and hydrogel sensing element.

### Sensitivity Analysis

The sensitivity is defined as the change of the output signal in relation to the change of the input signal, hence,
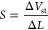
1Thereby, Δ*V*_st_ denotes the change of the steady-state voltage due to the stimulus concentration, and Δ*L* indicates the amount of change in the stimulus level (Δ*C* for glucose, ΔpH for pH). Note that there are several definitions for the sensitivity of a sensor in literature.^65^ Here we refer to sensor sensitivity as the output signal change for a given change in the measured quantity.

The steady-state voltage (and corresponding standard deviation) is calculated as the average of the last 2 h of each solution exchange interval (corresponding data and details are listed in the Supporting Information). The sensitivity has been calculated for each stimulus change from step and reset test data (Figure 4c–f). As there are four corresponding values for each change (e.g., for GSH a step from 0 to 3 mM occurs four times), these have been combined into average values for the same step.

**Figure 5:**
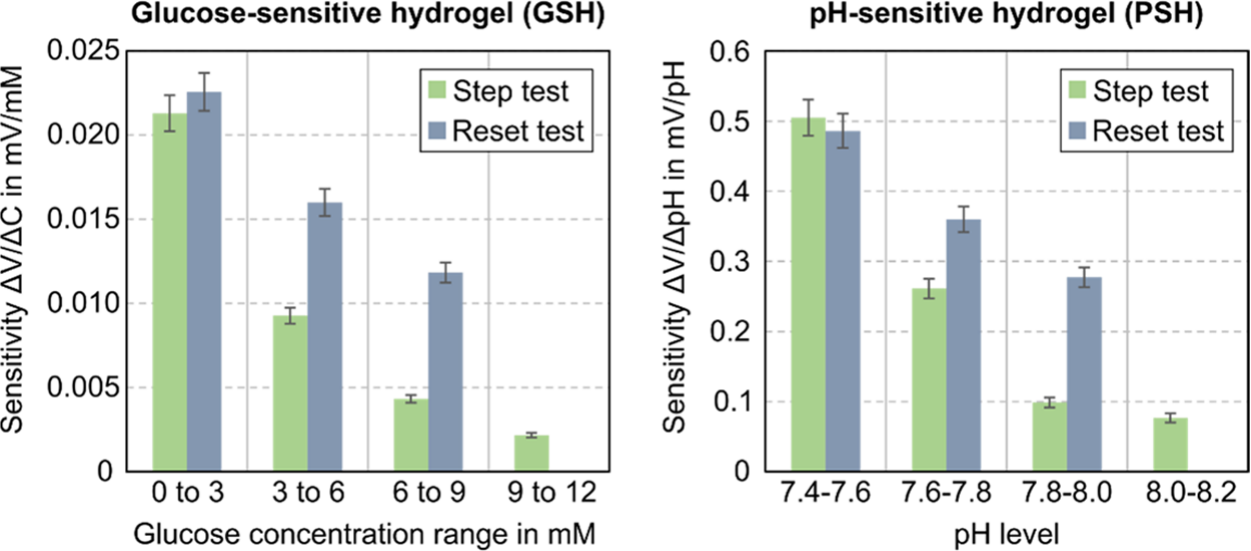
Sensitivities from step and reset tests for GSH and PSH, respectively. The values represent averages from 4 data points each, and the sensitivity has been calculated based on eq 1. The intervals in the *x*-axis represent the respective concentration/level step as shown in Figure 4b,c,e,f.

Figure 5 depicts the resulting sensitivities for GSH and PSH and step as well as reset test. In all cases, the trend of decreasing sensitivity with increasing stimulus concentration is visible, which is in accordance with the expected behavior of the hydrogel sensing element.^46,47^

Furthermore, the reset test consistently shows a higher sensitivity for the same concentration/level change as the step test. This can also be attributed to the hydrogel’s hysteretic behavior as the stimulus accumulates with ever increasing concentration in the step test and the corresponding reduced number of binding sites within the polymer network. In contrast, the immersion in pure PBS after each stimulus exposure in the reset test allows the material to recover to its initial state.

From these observations, it can be concluded that the sensitivity is dependent on the type of sensing material and governed by its hysteretic behavior. From the perspective of the transduction concept, it can be stated that it is capable of fully tracking the sensing material’s swelling behavior in all aspects.

### Limit of Detection

Based on the measurements described above, the limit of detection (LOD) for both sensors can be estimated. For the GSH, this denotes the minimum glucose concentration, and for the PSH the minimum change of pH that can be detected. The LOD determination follows the procedure outlined in ref (66), and details are described in the Supporting Information.

First, the standard deviation σ_b_ from repeated baseline measurements (5 times) is obtained from the functionality test, i.e., 0 mM for GSH and pH = 7.4 for PSH. Note that the steady-state voltage of each baseline measurement is already a mean value from the last two h of the measurement interval and therefore also has a standard deviation. However, this value is approximately 1 order of magnitude smaller than the variance of the five mean baseline values and therefore only the latter is considered for the LOD calculation.

In a second step, the sensor output signal is plotted with respect to the stimulus (concentration or change), and a linear regression fit is performed. The resulting slope *A* of the fitting curve is used to calculate the LOD:^66^
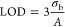
2For the sensor equipped with a GSH, the data from the step test are considered in the linear regression. Thereby, the mean values of the steady-state voltages for each studied glucose concentration of (0; 3; 6; 9; 12) mM are used, as described for the sensitivity characterization above. Since the linear range of hydrogel response with respect to glucose is limited approximately below 6 mM,^46,64^ only these are included in the regression fit (Figure S6 in Supporting Information). Note that there are different definitions of this specific hydrogel’s linear range, and it can also be considered up to 9 mM. However, we have chosen the conservative range of up to 6 mM where the hydrogel’s swelling response to glucose can be estimated as a linear dependence,^64^ since the focus is on the transduction principle and not on the sensing material performance.

For the PSH, the LOD is defined as the minimum pH-change that can be measured, and therefore, the data from the reset test are used in the linear regression fit as this comprises different pH-change steps (i.e., 0.2/0.4/0.6). Again, the average steady-state voltages are considered. The relevant quantities as well as the final values for LOD of both sensors are summarized in Table 1.

**Table 1:** Limit of Detection (LOD) Estimate for the Power-Transfer Concept with Glucose- and pH-Sensitive Hydrogel^a^

quantity	GSH	PSH
σ_b_ (funct. test)	0.00232 mV	0.00264 mV
regression	step test	reset test
slope	0.0153 mV/mM	0.5221 mV/ΔpH
*y*-intercept	0.1766 mV	0.5822 mV
*R*^2^	0.95	0.99
LOD	0.5 mM	0.02 ΔpH

a The baseline standard deviation σ_b_ is obtained from 5 measurements from the functionality test, and the linear regression fit is performed for step (GSH) and reset (PSH) test data.

Note that the sensor’s LOD is strongly dependent on the amount of hydrogel volume change for a given stimulus. In essence, the sensor’s total LOD can be considered as a combination of (i) the transducers’ ability to respond to minimal changes in the hydrogel’s swelling state close to the minimum values of the parameter (e.g., analyte) of interest and (ii) the amount of the hydrogel’s swelling change at this point.

A more general LOD estimate for the presented transduction principle could therefore be based on relating the observed volume change of the hydrogel to the sensor output voltage, regardless of the specific stimulus and its strength that cause the volume change. However, with the flow-cell setup used in the presented measurements, it is not possible to integrate optical swelling observation with a microscope, due to the required electromagnetic shielding and limited observation angles. The hydrogel would only be visible from the side, and it is very difficult to extract reliable information about the amount of volume change from such a limited perspective. Therefore, we opted for calculating the LOD based on the specific stimulus to achieve a much more robust estimate.

In general, to lower the LOD, either a new hydrogel material with an increased absolute swelling response close to the analyte/parameter change minimum can be designed or the transducer can be modified to be more sensitive to smaller hydrogel volume changes. The latter could, for example, be achieved by using thinner PI layers or adjusting the shape of the metal strip line. However, this can also result in an overly strong deformation of the sensor at higher stimulus levels or increased noise, limiting the sensing range. Please note that these considerations also apply to the sensitivity as this is also dependent on both the transducer and the sensing material.

Finally, the presented calculations of the LOD enable a conclusion about the transducer structure: The transducers employed for both test analytes are identical except for variations due to sensor assembly. In the baseline measurements, i.e., at 0 mM glucose and pH = 7.4, respectively, it can be assumed that the hydrogel is in its initially fabricated state and therefore has a similar target thickness (400 μm defined by mold) on both sensors. Comparison of the baseline’s standard deviations (σ_b_) for the GSH and PSH sensor indicates a very good stability and reproducible properties of the transducers, since these values only differ by 0.3 mV.

The proof-of-concept studies described above clearly indicate the feasibility of the power transfer-based transduction principle for smart hydrogel swelling state detection and have been demonstrated for two different hydrogel sensing elements and stimuli. These investigations have been carried out with a test geometry designed to minimize additional influences such as mechanical instabilities due to low PI film stiffness and increased noise/interferences and resistance due to longer metal strip lines. The advancement of the transduction principle toward the envisioned catheter integration certainly requires comprehensive design considerations of mechanical and electrical influences and properties, and likely several optimization iterations.

## Conclusion and Outlook

Enhancing the functionality spectrum of microcatheters by integrating additional sensing capabilities offers a promising way for acute blood analyte monitoring, e.g., during surgical procedures. However, such developments are currently sparse due to the challenging requirements in a biomedical context and the lack of suitable sensor approaches and sensing materials for specific target analytes. One approach to implement such sensors is by using stimulus-responsive (i.e., smart) hydrogels as an easily adjustable, sensitive, and selective sensing material for analyte detection.

However, a persistent challenge that hinders harnessing the stimulus-dependent swelling change of the hydrogel so far is the lack of suitable transduction concepts to extract an electrical signal from the hydrogels’ volume change. The presented study aims at providing a proof-of-principle verification of a novel power transfer-based concept for this purpose.

To this end, the swelling responses of two different smart hydrogels (glucose- and pH-sensitive) were studied by using different test protocols. Thereby, the stimuli were either alternated between two levels or increased and decreased in a stepwise fashion with or without resetting of the material after each step. A consistent behavior in accordance with the expected hydrogel response, and a very good stability and reproducibility were found. Further analysis of sensitivity and limit of detection shows that hydrogel thickness changes in the single-figure percentage range can be reliably detected.

The core functional principle is based on power transfer between a sending and a receiving transducer part with no influence from the specific hydrogel except for providing the stimulus-dependent actuation. Therefore, any type of smart hydrogel composition and stimulus that lead to the hydrogel’s volume change can be used as a chemical sensing element as long as they can be securely attached to the transducer material. Additionally, the transducer is only made from materials that are commonly used in biomedical contexts and are considered biocompatible at least for acute applications.^40^

Hence, from a material perspective, the developed sensor concept is suitable for integration into a microcathetert for blood analyte monitoring. In view of this target application, the transducer shape and dimensions for the presented proof-of-concept studies are designed to fit into a standard medical catheter without obstructing its normal use. Future developments will focus on creating a fully functional smart catheter and demonstrating its *in vivo* viability. This will encompass design advancements of the transducer as well as hydrogel engineering with regard to its swelling behavior to ultimately enable real-time and in situ biomedical analyte monitoring.

## Supporting Information Available

Detailed description of transducer fabrication process; hydrogel precursor solution preparation; sensor assembly; frequency spectrum of sensor; solution exchange protocol; data for control sensor; steady state voltages; limit of detection calculation (PDF)
