# Supplementary material for: Smart Hydrogel Swelling State Detection Based on a Power-Transfer Transduction Principle
Source: ACS Appl Polym Mater. 2024 Apr 23;6(9):5544–54. doi: 10.1021/acsapm.4c00808 (PMC11091848; doi:10.1021/acsapm.4c00808)
Supplement: Supplementary file 1 — ap4c00808_si_001.pdf [file ap4c00808_si_001.pdf]

Supporting Information

Smart Hydrogel Swelling State Detection Based on a Power-Transfer Transduction Principle

Benozir Ahmed[1], Christopher F. Reiche[1], Jules J. Magda[2], Florian Solzbacher[1], Julia Körner [3, *]

[1] Department of Electrical & Computer Engineering, University of Utah, Salt Lake City, UT 84112 USA
[2] Department of Chemical Engineering, University of Utah, Salt Lake City, UT 84112 USA
[3] Faculty of Electrical Engineering & Computer Science, Leibniz Universität Hannover, 30167 Hannover, Germany
* Correspondence: koerner@geml.uni-hannover.de

Table of Contents:

1. Transducer fabrication
2. Preparation of precursor solutions
3. Sensor assembly
4. Frequency spectrum of the sensor
5. Solution exchange protocol
6. Control sensor data
7. Steady-state voltage plots
8. Limit of detection calculation
9. References

1. Transducer fabrication

The complete power transfer-based sensors were created in two steps: i) microfabrication of the individual polyimide (PI) encapsulated conductive transducer structures (Figure S1), and ii) assembly of two of these transducer parts with a smart hydrogel sensing element and electromagnetic (EM) shielding as shown in Figure S3.

For the transducer structures, first liquid polyimide precursor (PI 2611) is spin-coated on a silicon carrier wafer, followed by a soft and a hard bake in nitrogen atmosphere (Figure S1a). Next the metal structures are created by a two-layer photoresist photolithography and lift-off process: lift-off resist (LOR) and positive tone photoresist (S1813) are used as the bottom and top layer respectively. A mask aligner and chrome mask are used to expose the photoresist and the structure are developed using AZ 300 MIF developer (Figure S1b). Afterwards a Ti/Pt layer stack (approximately 10 nm Ti and 790 nm Pt) is deposited by sputtering and the conductive structures are finally created by a subsequent lift-off step in an ultrasonication bath in acetone (Figure S1c). Then, another layer of PI is spin-coated onto the wafer and again subjected to soft and hard backing steps for encapsulation of the metallic traces (Figure S1d). The outline of the sensor structures and the bondpads are created by a second photolithography step with AZ 9260 photoresist and AZ 400K developer. A deep reactive ion etch (DRIE) process with CF_4, O_2, and N_2 gases is performed to remove polyimide from the bondpads and etch through to the wafer at the sensor outline (Figure S1e and Figure S1f). Thereby, the photoresist is used as the etch mask and the metal acts as a hard mask to prevent further etching of the PI at the bondpad site. Finally, the carrier wafer with sensors is rinsed with acetone, IPA and deionized (DI) water to remove the photoresist residue. An image of the final transducer structure is depicted in Figure S2 and a detailed account of the used fabrication parameters, chemicals and equipment is provided in Table S1.

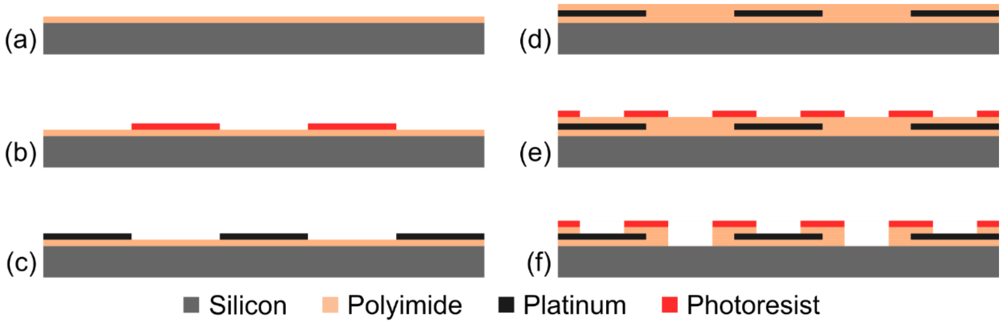

Figure S1. Schematic of individual polyimide (PI) encapsulated platinum structure fabrication process: (a) liquid PI is spin-coated on the carrier substrate (silicon) and baked to solidify. (b) Patterning of photoresist for the definition of metal traces. (c) The platinum structure is generated by sputter deposition and a subsequent lift-off process. (d) Another layer of PI is deposited to encapsulate the platinum structure. (e) Patterning of photoresist to use as a etch mask. (f) Dry etching of the PI to access the platinum bondpads and define the sensor outline.

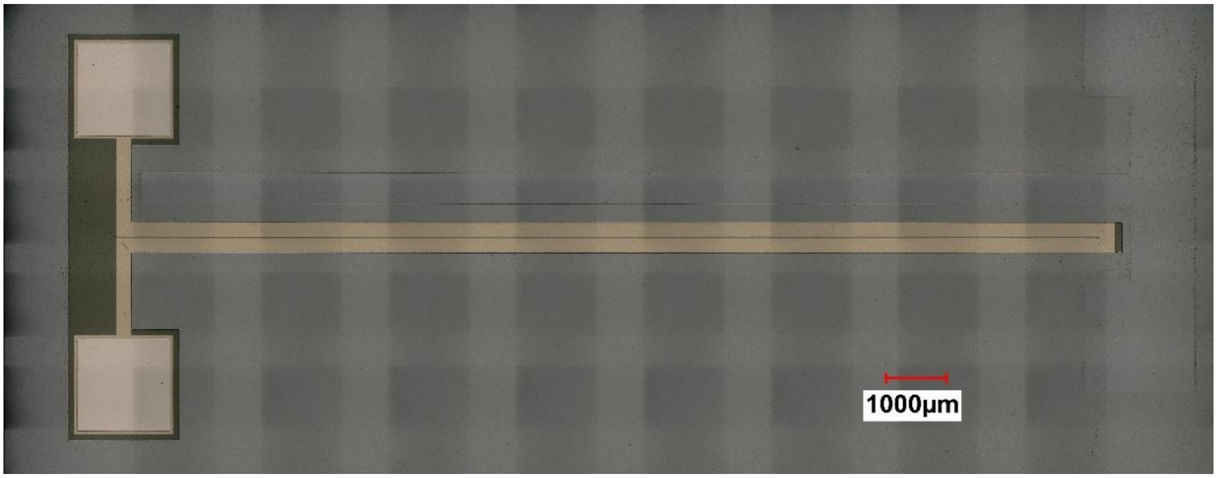

Figure S2: Completed microfabricated transducer structure. Please note that sensors are rather large. Therefore, the microscope's stitching feature was used to create the image, resulting in the striped contrast pattern artifacts.

Table S1. Detailed process information for fabrication of individual transducer parts comprising metal traces and a polyimide encapsulation.

Step #	Process Step	Process parameter	Equipment	Manufacturer
1	PI coating	Chemical: PI 2611<br>Spin speed: 4500 RPM<br>Soft bake: @130°C for 90s<br>Hard bake in nitrogen environment (80 mmHg pressure): 2-hour temperature ramp from RT to 300°C, 1 hour rest at 300°C, 1 hour ramp down to RT	CEE 200X 1800 Spinner	Spinner: Brewer Science, USA;<br>PI 2611: HD MicroSystems, USA
2	1st lithography (bottom layer)	Photoresist: LOR 10B<br>Spin speed: 1500 RPM<br>Soft bake: @180°C for 300s<br>Hard bake: N/A	CEE 200X 1800 Spinner	Spinner: Brewer Science, USA;<br>LOR 10B: MicroChem, USA
3	1st lithography (top layer)	Photoresist: S1813<br>Spin speed: 2000 RPM<br>Soft bake: @110°C for 60s<br>Hard bake: N/A	CEE 200X 1800 Spinner	Spinner: Brewer Science, USA;<br>S1813: Microposit, USA
4	Exposure	Dose: 60 mJ/cm^2	SUSS MA 1006	SUSS MA 1006: SUSS Micro Tec, USA
5	Development	Time: 45 s	Chemical: AZ 300 MIF developer	AZ 300 MIF: AZ Electronic Materials
6	Pt/Ti sputtering	Environment: Argon (150 sccm)<br>Ar pressure: 10 mTorr<br>DC power: 50 W	TM Vacuum Sputter System	Sputter system: TM Vacuum Products, USA
7	Lift-off	Time: 30 minutes (ultrasonication)	Chemical: Acetone	Acetone: PureSolv, USA
8	PI coating	Chemical: PI 2611<br>Spin speed: 4500 RPM<br>Soft bake: @130°C for 90s<br>Hard bake in nitrogen environment (80 mmHg pressure): 2 hour temperature ramp from RT to 300°C, 1 hour rest at 300°C, 1 hour ramp down to RT	CEE 200X 1800 Spinner	Spinner: Brewer Science, USA
9	2nd lithography	Photoresist: AZ 9260 (double layer)<br>Spin speed: 1100 RPM<br>Soft bake: @110°C for 180s<br>Hard bake: N/A	CEE 200X 1800 Spinner	Spinner: Brewer Science, USA;<br>AZ9260:AZ Electronic Materials USA
10	Rehydration	Time: 1 hour in water vapor chamber @ room temperature	Rehydration bath	Custom made
11	Exposure	Dose: 400 mJ/cm^2	SUSS MA 1006	SUSS MA 1006: SUSS Micro Tec, USA
12	Development	Time: 10 minutes	Chemical: AZ 400K developer	AZ 400K: AZ electronic materials, USA
13	DRIE	Gases: CF4 (3 sccm), O2 (30 sccm), N2 (10 sccm)<br>RF power: 200 W, ICP power: 1000 W<br>Chuck Temperature: 0°C	Oxford 100 ICP	Oxford 100 ICP: Oxford Instruments, UK
14	Cleaning	Acetone, IPA, DI water		Acetone: PureSolv, USA;<br>IPA: J.T. Baker Chemical Co., USA

2. Preparation of precursor solutions

Glucose-sensitive hydrogels

For preparation of the precursor solution for the glucose sensitive hydrogel (GSH), the recipe described in the work of Farhoudi et. al [1] is used. In summary, the following steps were conducted: A 1 mM buffer solution of 4-(2-hydroxyethyl)piperazine-1-ethanesulfonic acid (HEPES, Sigma-Aldrich) is prepared by dissolving HEPES powder in deionized (DI) water. By adding of 1 M HCl or NaOH, respectively, the pH of the buffer solution is adjusted to 8.0 at 20°C. Next, an appropriate amount of acrylamide (Fisher Scientific) is added to this 1 mM HEPES buffer to obtain a 30% w/w monomer solution. Following that, 19.1 mg of the anionic monomer 3-acrylamidophenylboronic acid (3APB, Frontier Scientific, Inc) is dissolved in 87 µL of dimethyl sulfoxide (Sigma-Aldrich) and then mixed with 237 µL of the 30% w/w solution of acrylamide in 1 mM HEPES buffer, 193 µL of 2% w/w solution of the crosslinker N,N′-methylenebisacrylamide (Sigma-Aldrich), 309 µL of 1 mM HEPES buffer, and 20.48 µL of the cationic monomer N-[3-(dimethylamino)propyl]methacrylamide (Sigma-Aldrich), in that order. Finally, 25.8 µL of 4% w/w solution of photoinitiator lithium phenyl-2,4,6-trimethylbenzoylphosphinate (LAP, Sigma-Aldrich) in 1 mM HEPES is added to the monomer solution.

pH-sensitive hydrogels

For preparation of the precursor solution for the pH sensitive hydrogels (PSH), first 1 mM buffer solution of 4-(2-hydroxyethyl)piperazine-1-ethanesulfonic acid (HEPES, Sigma-Aldrich) is prepared by adding 47.66 mg HEPES powder to 195 mL of deionized (DI) water. The pH of the buffer is adjusted to 8.0 at 20 ˚C by adding the required amount of either 0.1 M HCl or NaOH. Then, 4% w/w solution of the photoinitiator lithium phenyl-2,4,6-trimethylbenzoylphosphinate (LAP, Sigma-Aldrich) is prepared by adding 40 mg of photoinitiator powder to 960 µL of 1 mM HEPES solution.

To create a 30% w/w monomer solution, 15 g acrylamide (Fisher Scientific) is added to 35 mL of 1 mM HEPES solution. Then, 18.3 mg of 3-acrylamidophenylboronic acid (3APB, Frontier Scientific, Inc) is dissolved in 87 µL of dimethyl sulfoxide (Sigma-Aldrich) and combined with 237 µL of the 30% w/w acrylamide solution in 1 mM HEPES buffer. In turn, 193 µL of a 2% w/w solution of the crosslinker N,N′-methylene-bisacrylamide (Sigma-Aldrich), 309 µL of 1 mM HEPES buffer, and 47.4 µL of N-[3-(dimethylamino)propyl]methacrylamide (Sigma-Aldrich) are sequentially added to the mixture. Furthermore, the 25.8 µL of the already prepared 4% w/w solution of LAP photoinitiator solution is added to the solution.

3. Sensor assembly

To fully assemble the sensors, first the microfabricated transducer structures are peeled off from the silicon carrier wafer individually with tweezers. Two of them are glued to the electromagnetic shield in a back-to-back configuration with the shield in-between utilizing medical grade epoxy (Figure S3a). The shield is made from a 1 mm thick glass substrate sputter coated with a Cr/Au layer (10 nm chromium, 190 nm gold) on both sides.

Cables are connected to the bondpads by soldering, and subsequently the connection points are reinforced and encapsulated with medical grade epoxy (see Figure S3b). Aluminum foil shields sputter coated with 200 nm Cr/Au (10 nm chromium, 190 nm gold) are attached to the other side of the PI films to reduce the impact of external electromagnetic interference (Figure S3c).

One potential failure mode for this sensor is the delamination of hydrogel from the PI surface. In order to securely attach the hydrogel, the PI surface is pre-treated using oxygen plasma and further chemically treated using methanol, aminopropylmethacrylamide·HCl and distilled

tributylamine [2]. This improves the adhesive between sensor surface and hydrogel prior to polymerization.

Next, the smart hydrogels are fabricated between the PI transducer parts tips with an UV polymerization technique. In order to control the thickness of the hydrogel, a 400 µm thick polytetrafluoroethylene (PTFE) mold is additionally used (Figure S3d). The precursor is filled into the mold and then exposed to UV light of 395 nm wavelength for 30 seconds (approximate dose 13 J/cm²) to achieve polymerization. After polymerization, the sensors are thoroughly washed in DI water to remove the unpolymerized precursor solution. Finally, the hydrogel is conditioned alternating in 1x phosphate-buffered saline (PBS) and 1/3x PBS for 8 cycles with 8 hours of solution exchange interval.

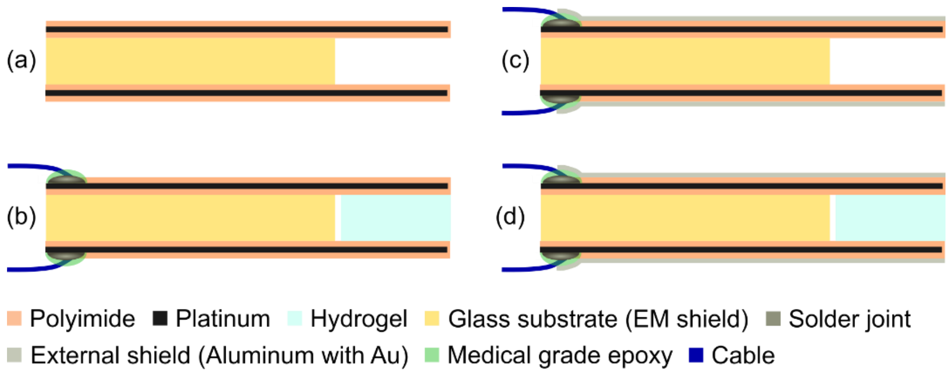

Figure S3. Schematic of power transfer-based smart hydrogel sensor assembly (a) two PI encapsulated metal structures are connected back-to-back with a gold coated glass substrate acting as an electromagnetic shield in-between is used. (b) Cables are connected to the bondpad via soldering and subsequently encapsulated with medical grade epoxy, (c) finally everything is covered with a gold sputtered aluminum shield except for the sensor tip, (d) hydrogel is polymerized between the tips of the PI structures with mold-based UV polymerization.

4. Frequency spectrum of the sensor

The operating frequency for the time-domain signal recording during different tests (functionality, step, reset test) is determined by recording a frequency spectrum of the power transfer-induced voltage on the receiver side within the (1-100) MHz frequency range. This is accomplished by using a lock-in amplifier (Zurich Instruments UHFLI, Switzerland). The initial frequency spectrum is recorded while the flow cell containing the sensor is filled with 1x PBS. Next, the solution is exchanged to a 5 mM glucose solution (in 1x PBS buffer) by continuous flow through a programmable syringe pump (Model 780212, KD Scientific Inc.). Another frequency spectrum is recorded after 6 hours of sensor immersion in the continuous solution flow when the hydrogel has reached an equilibrium.

Based on comparison of the obtained frequency spectra, the maximum change of induced voltage is found at ~13 MHz (Figure S4). Therefore, 13 MHz (closest full number to the resonant frequency) was chosen as the operating frequency for the time-domain tests with the sensor.

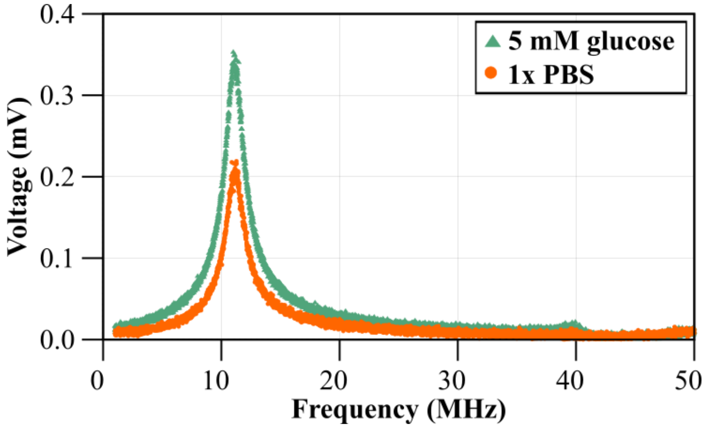

Figure S4. Power transfer-induced voltage on the receiver side when the sensor is immersed in 1x PBS and 5 mM glucose solution, respectively. The maximum change is observed at ~13 MHz.

5. Solution exchange protocol

Table S2. Solution exchange protocol for different experiments with glucose- and pH-sensitive hydrogel (GSH, PSH respectively).

Step no.	Functional. test		Step test		Reset test	
	GSH (mM)	PSH (pH)	GSH (mM)	PSH (pH)	GSH (mM)	PSH (pH)
initial	0	7.4	0	7.4	0	7.4
1	5	7.7	3	7.6	3	7.6
2	0	7.4	6	7.8	0	7.4
3	5	7.7	9	8.0	6	7.8
4	0	7.4	12	8.2	0	7.4
5	5	7.7	9	8.0	9	8.0
6	0	7.4	6	7.8	0	7.4
7	5	7.7	3	7.6	3	7.6
8	0	7.4	0	7.4	0	7.4
9	-	-	3	7.6	6	7.8
10	-	-	6	7.8	0	7.4
11	-	-	9	8.0	9	8.0
12	-	-	12	8.2	0	7.4
13	-	-	9	8.0	-	-
14	-	-	6	7.8	-	-
15	-	-	3	7.6	-	-
16	-	-	0	7.4	-	-

6. Control sensor data

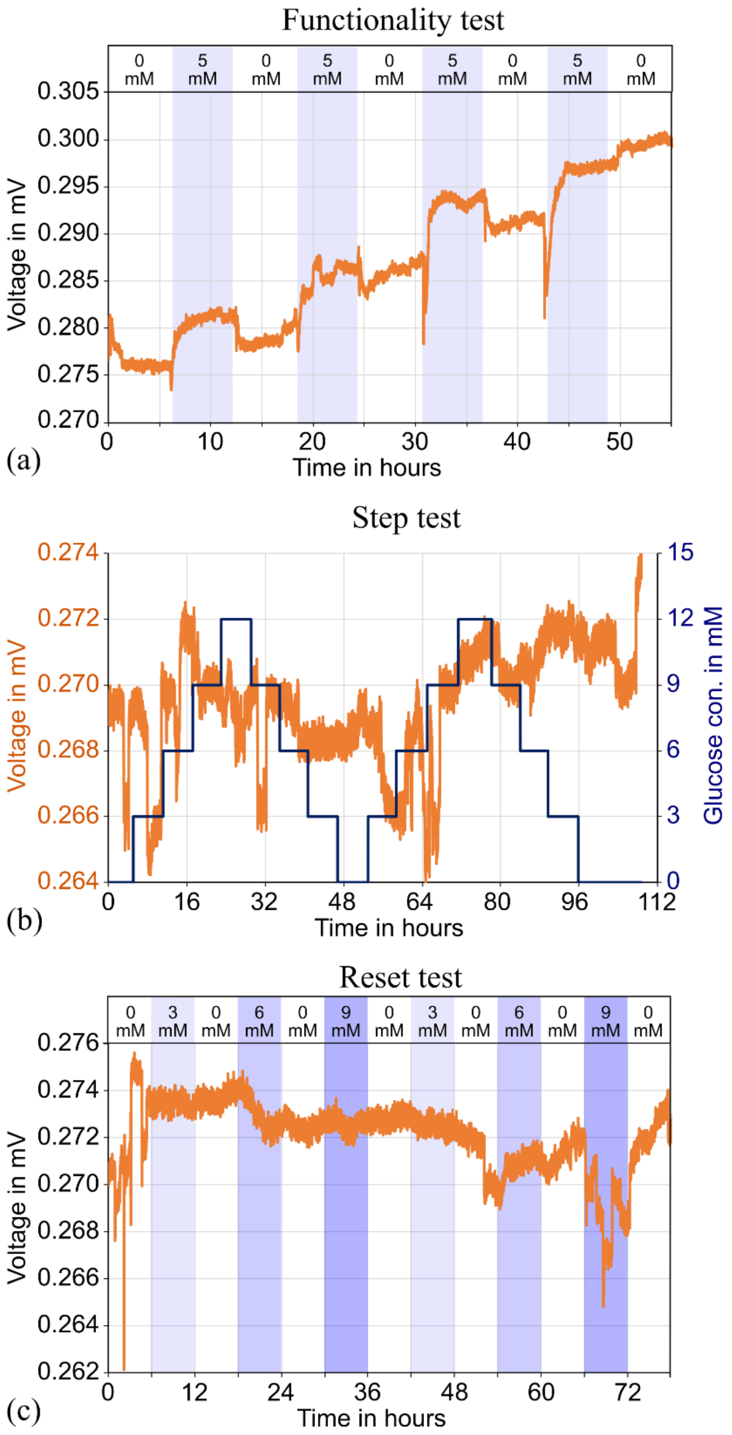

Figure S5. Results of (a) functionality, (b) step and (c) reset test with varying glucose concentrations for a non-responsive control sensor where medical grade epoxy was used instead of a smart hydrogel. All test conditions as well as the dimensions and shape of the epoxy were the same as for the functional sensors.

7. Steady-state voltage plots

Table S3. Steady-state voltages for glucose-sensitive hydrogel (GSH) for each concentration in step and reset test. The values and corresponding standard deviations (StdDev) are calculated as averages from the last two hours of data for each step.

GSH	Step test		Reset test	
Glucose in mM	Stead. Volt. in mV	StdDev in mV	Stead. Volt. in mV	StdDev in mV
0	0.170793	1.51E-04	0.169663	6.20E-05
3	0.233894	1.04E-04	0.238201	1.19E-04
6	0.261043	8.37E-05	0.169526	4.92E-05
9	0.275343	2.24E-04	0.266749	1.05E-04
12	0.268975	2.92E-04	0.169775	6.48E-05
9	0.275222	1.18E-04	0.277003	9.84E-05
6	0.262687	1.48E-04	0.172264	7.20E-05
3	0.234393	1.29E-04	0.238570	8.94E-05
0	0.170840	6.11E-05	0.171318	6.04E-05
3	0.234314	9.38E-05	0.266326	1.34E-04
6	0.262354	9.04E-05	0.171579	7.26E-05
9	0.275271	8.43E-05	0.278428	1.20E-04
12	0.268638	9.14E-05	0.171698	7.38E-05
9	0.275514	1.04E-04		
6	0.263337	9.50E-05		
3	0.235544	8.63E-05		
0	0.170168	7.27E-05		

Table S4. Steady-state voltages for pH-sensitive hydrogel (PSH) for each level in step and reset test. The values and corresponding standard deviations (StdDev) are calculated as averages from the last two hours of data for each step.

PSH	Step test		Reset test	
pH	Stead. Volt. in mV	StdDev in mV	Stead. Volt. in mV	StdDev in mV
7.4	1.270693	6.01E-04	1.245450	5.20E-04
7.6	1.369108	9.06E-04	1.343643	6.31E-04
7.8	1.414109	6.23E-04	1.245539	6.07E-04
8.0	1.434810	6.79E-04	1.390651	1.06E-03
8.2	1.450948	1.08E-03	1.246564	5.70E-04
8.0	1.435500	6.61E-04	1.410844	5.85E-04
7.8	1.417733	1.33E-03	1.247747	5.26E-04
7.6	1.345515	6.41E-04	1.344142	5.47E-04
7.4	1.243844	6.19E-04	1.247906	5.48E-04
7.6	1.344864	5.33E-04	1.389268	5.07E-04
7.8	1.391797	5.65E-04	1.243904	5.10E-04
8.0	1.412521	5.14E-04	1.411242	7.06E-04
8.2	1.427939	6.22E-04	1.240396	6.30E-04
8.0	1.413678	6.03E-04		

7.8	1.393714	5.79E-04		
7.6	1.348773	5.17E-04		
7.4	1.246061	6.11E-04		

8. Limit of detection calculations

The determination of limit of detection (LOD) is based on the procedure outlined in [3]:

(i) Calculation of standard deviation σ_b of repeated baseline measurements.
In the presented case, the five measurements in 0 mM glucose (for GSH) and pH = 7.4 (for PSH) from the functionality test are used (shaded rows in table S5). Thereby, the steady-state voltages with standard deviation summarized in table S5 have been obtained as mean values from the last two hours of each concentration. For LOD calculation, the standard deviation of these five mean values has been considered as the individual standard deviations from the two hour averages are approximately one order of magnitude smaller.

(ii) Linear regression fit of sensor output voltage with respect to stimulus
The mean values of the steady-state voltages from step test (GSH) and reset test (PSH) are plotted over the stimulus concentration, which is either the absolute glucose amount or the change of pH level (figure S6). Again, these mean values are averages from (i) the last two hours of each measurement interval and (ii) from four data points of the same concentration or step height as outlined for the sensitivity calculation in the main text.

A linear regression fit is performed for each data set and the resulting slope A of the regression line used to calculate the LOD with [3,4]:

$$LOD = 3 \cdot \frac{\sigma_b}{A} \quad \text{(eq. S1)}$$

The values for each fit are shown in the graphs in figure S6 and the resulting LOD are 0.5 mM for the glucose sensor and 0.02 ∆pH for the pH-sensitive one.
Please note that for the GSH, only the first 3 data points are considered in the fit as only they are within the previously observed linear range of the used GSH [2].

Table S5. Steady-state voltages (averages of two hours) for glucose and pH-sensitive hydrogel for each level in functionality and step (GSH) or reset (PSH) test. For step and reset test, these 2 hour means have in turn been used to calculate an average of four values as each concentration / step occurred four times in one experiment (refer to figure 4 in main text). Only the shaded rows have been used to calculate standard deviation of the baseline measurements for each sensor. For GSH, the full data is listed for the step test but due to the limited linear range of the hydrogel, only the first three values have been considered in the fit.

**Glucose-sensitive hydrogel (GSH)**				
Functionality test (mean values)			Step test (mean values)	
Gluc. conc. in mM	Stead. Vol. In mV	StdDev in mV	Gluc. conc. in mM	Stead. Volt. in mV
0	0.169	0.000526	0	0.171
5	0.340	0.007972	3	0.235
0	0.173	0.000685	6	0.262
5	0.330	0.003142	9	0.275
0	0.170	0.000612	12	0.269

5	0.340	0.000703		
0	0.177	0.000549		
5	0.348	0.000595		
0	0.173	0.000589		
**StdDev σ_b (mV)**	**0.00232**			
**pH-sensitive hydrogel (PSH)**				
Functionality test (mean values)			Reset test (mean values)	
pH	Stead. Vol. In mV	StdDev in mV	ΔpH	ΔStead. Volt. in mV
7.4	1.267	0.000526	0.2	0.486
7.7	1.407	0.000697	0.4	0.360
7.4	1.276	0.000685	0.6	0.277
7.7	1.406	0.001142		
7.4	1.273	0.000612		
7.7	1.402	0.000703		
7.4	1.269	0.000549		
7.7	1.400	0.005950		
7.4	1.270	0.000589		
**StdDev σ_b (mV)**	**0.00264**			

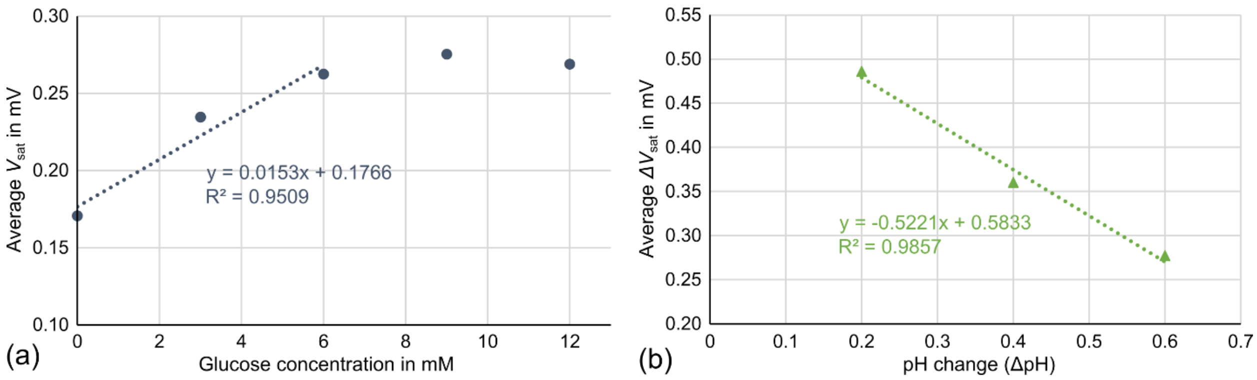

Figure S6. Linear regression fit of (a) step test data of GSH and (b) reset test data of PSH. For GSH, only the data points of the linear range of the hydrogel are considered in the fit but all data points are depicted for completeness. The resulting linear fit equations as well as the R^2 values are given in the graph.

9. References

[1] N. Farhoudi et al., ACS Sensors 5(7):1882, 2020; doi: 10.1021/acssensors.9b02180.
[2] S. Van Vlierberghe et al., Biomacromolecules 11(10):2731, 2010; doi: 10.1021/bm100783h.
[3] K Danzer: *Analytical Chemistry - Theoretical and Metrological Fundamentals*, Springer, Berlin Heidelberg, 2007.
[4] E. Voigtman: *Limits of Detection in Chemical Analysis*, John Wiley & Sons, 2017, p. 202.
